# Comparison of gradient concentration strip and EUCAST methods for itraconazole and posaconazole MICs in *Trichophyton indotineae*

**DOI:** 10.1128/aac.01293-25

**Published:** 2025-12-23

**Authors:** Brice Tireau, Samia Hamane, Stéphanie Weber, Mazouz Benderdouche, Sarah Wices, Alexandre Alanio, Sarah Dellière

**Affiliations:** 1 Laboratoire de Parasitologie-Mycologie, Assistance Publique-Hôpitaux de Paris, Groupe Hospitalier Saint-Louis-Lariboisière-Fernand-Widal 26934 , Paris, France; 2 Université de Paris 555089, Paris, France; 3 Translational Mycology Research Group, Mycology Department, Institut Pasteur, Université Paris Cité, National Reference Center for Invasive Mycoses and Antifungals 555089, Paris, France; 4 Institut Pasteur, Université Paris Cité, Immunology of Fungal Infection, Paris, France; University Children's Hospital Münster, Münster, Germany

**Keywords:** dermatophytes, *Trichophyton mentagrophytes* genotype VIII, resistance, Etest strips, itraconazole, posaconazole, *Trichophyton indotineae*

## Abstract

The increasing spread of antifungal-resistant dermatophytosis caused by *Trichophyton indotineae* has become a major public health and therapeutic concern. Consequently, antifungal susceptibility testing in routine clinical laboratories is essential for effective patient management. Itraconazole is currently the recommended treatment for these infections. However, few molecular or phenotypic tools are available to assess susceptibility to azoles. In this context, we evaluated the itraconazole and posaconazole MICs obtained using gradient concentration strips (GCS), in comparison with the EUCAST reference method. A total of 73 clinical isolates belonging to the *Trichophyton mentagrophytes* complex, including 64 *T. indotineae* isolates, were analyzed. MIC readings for both methods were performed on days 5 and 7 at partial (80%) and complete (100%) inhibition. We found that the optimal reading frame is on day 5 at 100% growth inhibition. Essential agreement within ±1 dilution (and ±2 dilutions) for the GCS method versus the EUCAST method was 65.8% (89%) for itraconazole and 57.5% (83.6%) for posaconazole. The GCS test appears to be a valuable method for susceptibility screening of *T. indotineae* clinical isolates, providing a practical option for routine laboratories despite essential agreement values below the ideal 90% threshold for method validation.

## INTRODUCTION

*Trichophyton indotineae*, corresponding to *Trichophyton mentagrophytes* genotype VIII, is an emerging dermatophyte species associated with recalcitrant and widespread dermatophytosis, particularly in the Middle East, India, and South Asia (1–3) and more recently in Europe and other parts of the world (4–7). Terbinafine (TBF), the primary drug used to treat dermatophytosis, has shown poor effectiveness due to a high prevalence of elevated minimum inhibitory concentrations (MICs) (8). This reduced efficacy has underscored the need to develop methods for assessing the susceptibility profiles of clinical strains, which were previously rarely performed for dermatophytes.

The study of the squalene epoxidase (SQLE) gene, the molecular target of TBF, has revealed several point mutations associated with high MICs *in vitro* (i.e*.,* F397L and L393S) (9, 10). Detection of these mutations enables the identification of isolates that are likely to be difficult to treat, given the absence of established clinical breakpoints for defining TBF resistance. To standardize the detection of TBF resistance, a reference method based on the European Committee on Antimicrobial Susceptibility Testing (EUCAST) has been developed for TBF and other antifungal agents (11, 12). While these methods demonstrate high inter- and intra-laboratory reproducibility, they are technically demanding, time-consuming, and require experienced personnel. As a more practical alternative, TBF gradient concentration strips (GCS) have been evaluated (13, 14). However, their clinical utility has been questioned due to batch-to-batch variability (13). Agar-screening method for TBF and itraconazole (ITZ) has also been evaluated. However, testing did not include any itraconazole-resistant isolates (15).

Given that TBF resistance rates can reach up to 80% (1, 2, 16), ITZ, an azole-class antifungal, is currently recommended as the first-line treatment, while posaconazole (PCZ) has shown promising results in small series (17–20). However, ITZ treatment failure has been reported in up to 47% of cases, raising concerns about its reliability (21). The cause of treatment failure is not yet fully understood. Potential contributing factors include poor skin penetration of ITZ due to suboptimal pharmacokinetics/pharmacodynamics with the dosing regimens used, poor adherence to therapy, recontamination from the environment, or elevated MICs (9, 22, 23). Nevertheless, numerous relapses have been documented even in cases where MICs were low (21). These findings highlight the importance of azole susceptibility testing. This is mainly based on the EUCAST method and molecular detection of mutations in the ERG11/CYP51 gene (24–26). However, neither approach is currently used in routine diagnostic practice. Moreover, in *T. indotineae*, elevated azole MICs have so far been attributed to overexpression of the *CYP51A*, rather than point mutations (26).

Commercial GCS for ITZ and PCZ are used for susceptibility testing of various non-dermatophyte fungi (27). However, their performance in dermatophytes remains underexplored. This study aims to (i) evaluate the performance of GCS for azoles, specifically for ITZ and PCZ, in comparison with the EUCAST reference broth microdilution method, for susceptibility testing of *T. mentagrophytes* complex isolates, including *T. indotineae* strains and (ii) determine whether SQLE genotypes are associated with specific azole MIC distributions.

## MATERIALS AND METHODS

### Strains

A total of 73 clinical isolates from 68 patients belonging to the *T. mentagrophytes* complex were selected from the Saint-Louis Hospital collection. Initial identification was based on macroscopic and microscopic morphology and subsequently confirmed by sequencing of the ITS region using primers LS266 (5′-GTATTCCCAAACAACTCGACTC-3′) and V9D (5′-TTAAGTCCCTGCCCTTTGTA-3′) (28). Among the 73 isolates, 64 were identified as *T. indotineae* (genotype VIII; GenBank accession MH791425.1). Based on *SQLE* gene mutations, their distribution was as follows: A448T (*n* = 23, 40.7%), F379L (*n* = 15, 25.4%), L393S (*n* = 9, 15.2%), wild type (*n* = 8, 13.5%), L393F (*n* = 1, 1.7%), F397L Y414H (*n* = 1, 1.7%), and A448T F397L (*n* = 1, 1.7%). These isolates were collected between 2017 and February 2025. Additional isolates collected between November 2024 and February 2025 included five *T. mentagrophytes* (genotype VII; GenBank accession MK447611.1, *n* = 4; genotype III*; GenBank accession; MK447604.1, *n* = 1) and four *T. interdigitale* (genotype II; GenBank accession MK447596.1, *n* = 4) (Table S1).

### Antifungal susceptibility testing

The inoculum was prepared by subculturing isolates on MALT agar (VWR chemicals, Belgium) for 9 days at 27°C. The microdilution broth method for dermatophytes was used to determine MICs of ITZ, PCZ, and TBF according to EUCAST recommendations based on 50% and 90% reduction in optical density after 5 days of incubation (11). Briefly, stock solutions of each antifungal were prepared at 1,600 µg/mL in dimethyl sulfoxide (DMSO), with final test concentrations ranging from 8 to 0.008 µg/mL in RPMI 1640 (BioMérieux; France) in 96-well plates and inoculated with conidia solutions at a final concentration of 3.3 × 10⁵ conidia. Plates were incubated at 27°C for 7 days and read spectrophotometrically at 490 nm at 5 and 7 days, using 90% and 50% inhibition endpoint.

In parallel, MICs using GCS were determined using Etest strips (BioMérieux, France) for PCZ and ITZ with MIC ranges of 0.002 μg/mL to 32 μg/mL. A conidia solution at 1 McFarland was inoculated into RPMI solid medium using a cotton swab. The swab was passed evenly over the RPMI medium, rotating 90° in three directions as recommended by the manufacturer. The GCS was placed on RPMI medium once the excess moisture had been absorbed. Plates were incubated at 27°C for 7 days. MICs were read at 5 and 7 days, at complete inhibition (no regrowth in the inhibition ellipse) and partial inhibition (without considering regrowth in the inhibition zone). Each isolate was tested once with both methods. In both methods, *Aspergillus fumigatus* reference strain ATCC 204305 was used for quality control.

### Interpretation of results and statistical analysis

To compare the results of the two methods used, MICs obtained with the strips were rounded up to the next dilution corresponding to the EUCAST concentrations. We then assessed the best correspondence between GCS and EUCAST method for ITZ and PCZ. This comparison was made based on four GCS MIC readings (complete 100% inhibition at 5 and 7 days, partial 80% inhibition at 5 and 7 days) and four broth microdilution MIC readings (90% inhibition at 5 and 7 days, 50% inhibition at 5 and 7 days). MIC values and differences were then compared using Student’s *t*-test, with a significance threshold set at *P* < 0.005. In exploratory analyses, MIC distributions for ITZ, PCZ, and TBF were stratified by SQLE genotype to assess potential lineage effects on method agreement.

## RESULTS

The median incubation period prior to antifungal susceptibility testing to obtain an important quantity of conidia was 9 days (interquartile range [IQR]: 7–13). The conidial concentration of the conidia solution was prepared to a target value of 1.0 McFarland for agar plate inoculation, corresponding to a median of 3.33 × 10⁶ conidia/mL (IQR: 2.4–4 × 10⁶).

**Fig 1:**
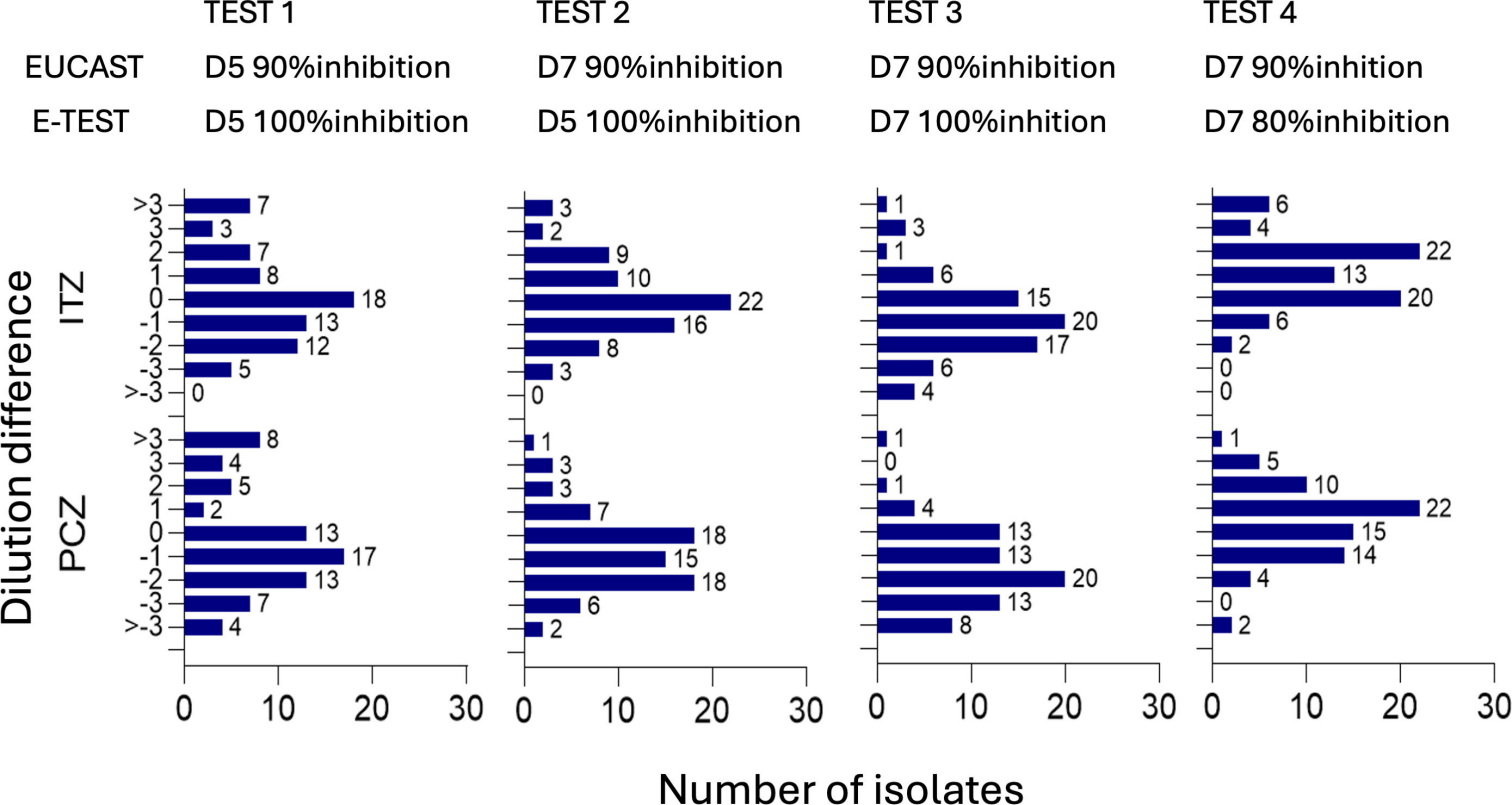
Distribution of isolates by MIC differences between EUCAST and GCS method for ITZ and PCZ. D : day ; ITZ : itraconazole ; PCZ : posaconazole.

**Fig 2:**
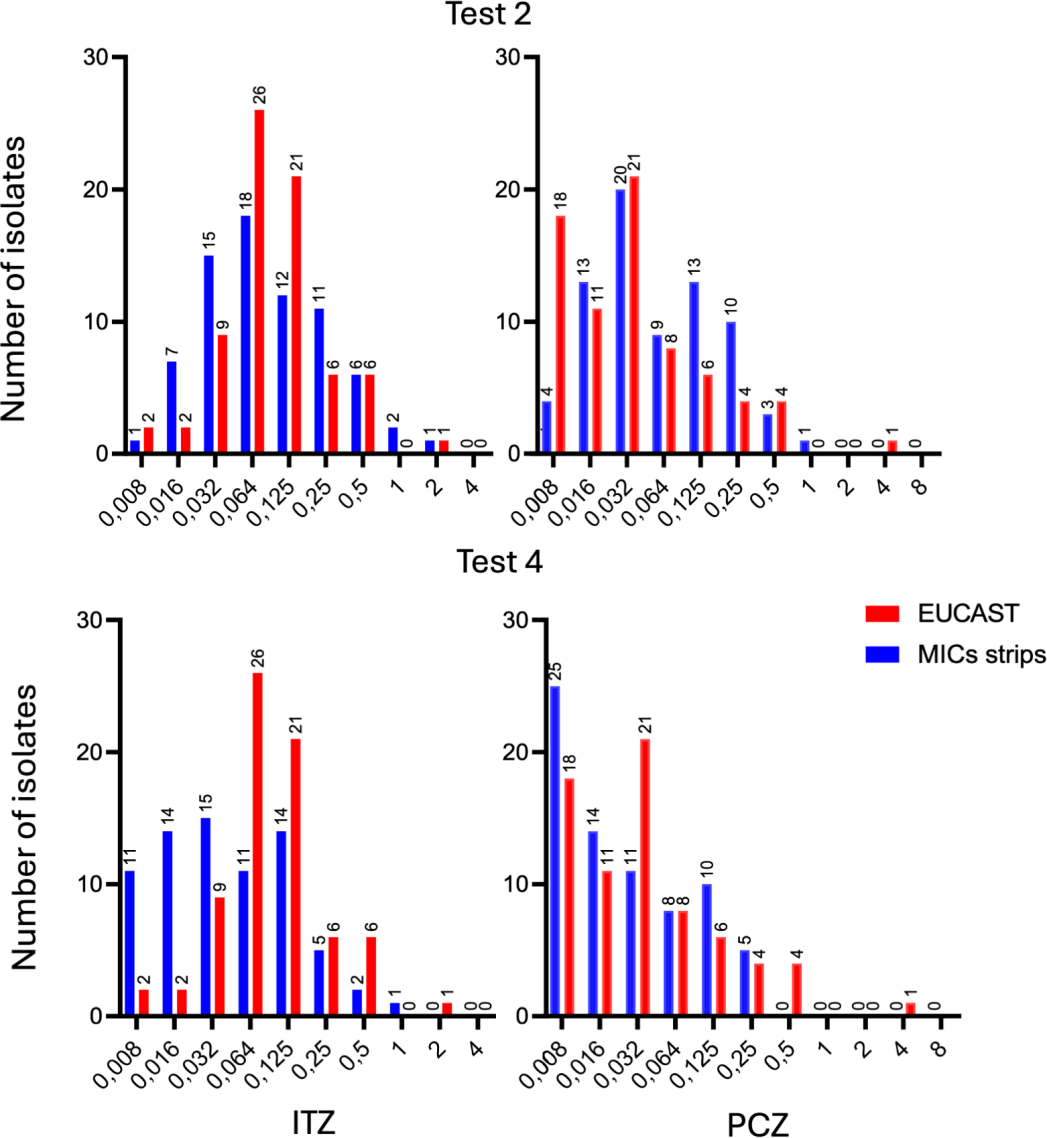
Distribution of EUCAST MICs and strips MICs isolates for tests 2 and 4.

Among the 16 comparisons between EUCAST (5 or 7 days, 50% or 90% inhibition) and GCS methods (5 or 7 days, 80% or 100% inhibition), four tests demonstrated the most favorable results, characterized by a near-Gaussian distribution of MIC differences and lowest observed mean and median values (Fig. 1; Table 1). The remaining 12 comparisons are detailed in Fig. S1. Optimal concordance between GCS and EUCAST MICs for both ITZ and PCZ was observed with two specific testing conditions: GCS MIC readings at D5 with 100% inhibition (test 2) and at D7 with 80% inhibition (test 4) (Table 1). Statistical comparison of MIC values between GCS and EUCAST for Test 2 showed no significant difference for either ITZ or PCZ (*P*-values: ITZ = 0.51, PCZ = 0.90). In contrast, Test 4 demonstrated a statistically significant difference in MIC values for ITZ (*P* = 0.01), but not for PCZ (*P* = 0.37). For Test 2, ITZ MICs ranged from 0.008 to 2 mg/L (median: 0.064, IQR: 0.032–0.25 mg/L) (Fig. 2). PCZ MICs ranged from 0.008 to 1 mg/L (median: 0.032, IQR: 0.032–0.125 mg/L). The essential agreement (EA) for Test 2 was 65.8/89.0 for ITZ and 57.5/83.6 for PCZ within ±1 and ±2-fold dilutions, respectively. Subsequently, the MIC distributions and corresponding EA stratified by SQLE mutation for ITZ, PCZ, and TBF are presented in Tables 2 and 3.

**TABLE 1:** Medians, means, and EA between GCS and EUCAST MICs

		Test 1	Test 2	Test 3	Test 4
EUCAST		Day 5–90%	Day 7–90%	Day 7–90%	Day 7–90%
GCS		Day 5–100%	Day 5–100%	Day 7–100%	Day 7–80%
ITZ^*a*^	Mean	0.42	0.24	−0.75	1.35
	Median	−0.02(IQR: −1.03/0.98)	0.0 (IQR: −1/0.98)	−0.99 (IQR: -1.60/–0.02)	1 (IQR: 0.40/1.98)
	% EA (±1/2 dilutions)	53.4/79.5	65.8/89	56.1/80.8	53.4/86.3
PCZ^*b*^	Mean	0	−1	−1	1
	Median	1 (IQR: −1.58/0.43)	−1 (IQR: −2/0)	−2 (IQR: −2.55/−0,05)	1 (IQR: −0.58/1.41)
	% EA (±1/2 dilutions)	49.3/71.2	57.5/83.6	41.1/69.9	69.9/89.0

^*a*^ ITZ, itraconazole.

^*b*^ PCZ, posaconazole.

**TABLE 2:** EA of GCS and EUCAST MICs according to SQLE genotype^*a*^

Genotype	ITZ		PCZ	
	±1 dilution	±2 dilutions	±1 dilution	±2 dilutions
A448T (*n* = 25)	61.5%	88.5%	60%	80%
F397L (*n* = 18)	57.9%	78.9%	38.9%	88.9%
L393S (*n* = 10)	60%	100%	80%	100%
WT (*n* = 8)	87.5%	87.5%	62.6%	87.5%

^*a*^ ITZ, itraconazole; PCZ, posaconazole; WT, wild type.

**TABLE 3:** Distribution of TBF, ITZ, and PCZ MICs for *T. indotineae* isolates according to SLQE mutations

		SQLE genotype						
		WT^*a*^	L393S	L393F	F397L Y414H	F397L	A448T	A448T F397L
TBF	0.008	1						
	0.016							
	0.032	3					6	
	0.064	3					15	
	0.125							
	0.25						2	
	0.5							
	1							
	2		3					
	4		6					
	8	1	1	1	1	18	2	1
ITZ	0.008	1					1	
	0.016		1				1	
	0.032	1	1			5		
	0.064	2	3		1	6	13	
	0.125	4	1	1		3	7	
	0.25		1			2	2	1
	0.5		3			1	1	
	1							
	2					1		
	4							
	8							
PCZ	0.008	2	3			6	4	
	0.016	2		1		3	5	
	0.032	3	1		1	4	9	
	0.064		2			1	4	
	0.125		1			2	2	
	0.25	1					1	1
	0.5		3			1		
	1							
	2							
	4					1		
	8							

^*a*^ WT, wild type.

Six isolates from five patients were follow-up samples collected after a median interval of 184 days (IQR, 106−227) following azole treatment regimens lasting 10 days to 8 weeks. Overall, azole MIC values remained stable over time in all five patients.

## DISCUSSION

The increasing use of azoles, particularly ITZ, for treating dermatophytosis caused by *T. indotineae* highlights the need for simple and reliable methods to detect antifungal resistance. In this context, GCS for ITZ and PCZ, already commercially available for other molds, may represent a practical option for azole susceptibility testing in dermatophytes. This study identified day 5 with 100% inhibition as the optimal reading condition, achieving EA within ±2 dilutions of the EUCAST method in 89% of cases for ITZ and 83.6% for PCZ.

Notably, neither trailing effect nor paradoxical growth, phenomena previously reported in other fungi, was observed for either antifungal agent tested (29–31). The absence of these effects reduces the likelihood of MIC reading errors when compared to the EUCAST reference method. Nevertheless, the visual interpretation of GCS remains a source of variability, with the accuracy of MIC interpretation dependent on the operator’s experience (32). Additionally, these azole GCS demonstrated reliable inter-batch reproducibility, in contrast to TBF GCS, for which marked lot-to-lot variability has been documented (13), further supporting their potential utility in routine susceptibility testing.

However, the implementation of the Etest method as a routine screening tool in clinical laboratory necessitates careful standardization of the inoculum, achieved by adjusting the suspension to a 1.0 McF turbidity. Among dermatophytes, *T. mentagrophytes* complex is well-suited for this approach due to its robust conidiogenesis, which is readily supported by standard culture media such as MALT. Conidial suspensions adjusted to 1.0 McF yielded a reproducible conidial concentration, with no significant variability observed across different days of harvest. In this context, inoculum preparation can be guided by visual assessment of fungal growth, relying on the expertise of the operator. Overall, the inoculum derived from 1.0 McF provided consistent and uniform growth on RPMI agar, enabling clear and interpretable inhibition ellipse formation with GCS.

Regarding the genotype-phenotype susceptibility profile, 25 isolates harbored the A448T substitution, previously associated with elevated ITZ MICs, although not directly responsible in targeted mutagenesis studies (1, 33, 34). We did not observe a correlation between A448T genotype and high azole MICs in our isolates. TBF MICs across different genotypes were consistent with existing literature (16). Finally, isolates cultured from patients previously treated with azoles did not show increased azole MICs, suggesting that relapse may be due to factors other than intrinsic resistance, such as host-related, pharmacokinetic issues, or recontamination. Two isolates from one patient, without additional *SQLE* mutations, exhibited variable TBF MICs (2–8 mg/L), further supporting the involvement of alternative resistance mechanisms, such as efflux pump overexpression (9). These findings highlight the value of combining phenotypic methods, such as GCS for azoles and TBF, with SQLE gene sequencing to improve resistance detection in routine diagnostics.

A key limitation of this study is the small number of isolates, particularly azole-resistant strains, which limits the ability to draw definitive conclusions regarding the EA of the two methods at higher MIC values. Further studies, including a greater number of azole-resistant isolates, are warranted. While the EA within ±2 dilutions reached 89% for ITZ and 83.6% for PCZ, below the 90% threshold generally expected for method validation (35), we consider these results still clinically relevant. GCS are not intended to replace broth microdilution methods but rather to serve as a practical screening tool for routine laboratories, enabling early detection of potentially resistant isolates. Another significant limitation, although beyond the scope of this study, is the inability to assess categorical agreement (CA), the classification of isolates as susceptible, intermediate, or resistant based on clinical breakpoints. CA is generally considered more clinically relevant than EA, as it directly informs therapeutic decision-making. However, clinical breakpoints for *T. indotineae* are currently unavailable, limiting the interpretation of MIC data and preventing the translation of laboratory findings into clinically actionable recommendations. The establishment of clinical breakpoints for *T. indotineae* is therefore an urgent priority, particularly in the context of rising antifungal resistance, treatment failure, and relapse. Until such breakpoints become available, interpretation of MIC data in guiding patient management remains limited.

### Conclusion

The GCS method (Etest) for azoles shows good agreement with the EUCAST reference method, especially when read on D5 with 100% inhibition. Standardization of the inoculum at 1.0 McF using conidial suspensions proved its reliability. This method offers a rapid and easy approach for routine susceptibility testing in clinical laboratories. Further validation is needed with additional isolates, particularly those exhibiting elevated MICs. However, the absence of established clinical breakpoints remains a major limitation for the clinical interpretation of MICs.

## SUPPLEMENTAL MATERIAL

Supplemental Material aac.01293-25-s0001.docx Table S1 and Figure S1.

ASM does not own the copyrights to Supplemental Material that may be linked to, or accessed through, an article. The authors have granted ASM a non-exclusive, world-wide license to publish the Supplemental Material files. Please contact the corresponding author directly for reuse.
