# Supplementary material for: Comparison of gradient concentration strip and EUCAST methods for itraconazole and posaconazole MICs in Trichophyton indotineae
Source: Antimicrob Agents Chemother. 2025 Dec 23;70(2):e01293-25. doi: 10.1128/aac.01293-25 (PMC12888879; doi:10.1128/aac.01293-25)
Supplement: Supplemental Material — Table S1 and Figure S1. [file aac.01293-25-s0001.docx]

**Table S1.** Characterization of isolates by genotype and SQLE gene mutations. NA: not assessed; WT: wild type

|  |  | *T. indotineae* | *T. interdigitale* | *T. mentagrophytes* | |
| --- | --- | --- | --- | --- | --- |
| Genotype |  | GVIII | GII | GVII | GIII* |
| *n* isolates |  | 64 | 4 | 4 | 1 |
| SQLE genotype | A448T | 25 |  |  |  |
|  | F397L | 18 |  |  |  |
|  | L393S | 10 |  |  |  |
|  | WT | 8 |  |  |  |
|  | L419F |  |  | 1 |  |
|  | A448T F397L | 1 |  |  |  |
|  | F397L Y414H | 1 |  |  |  |
|  | L393F | 1 |  |  |  |
|  | NA |  | 4 | 3 | 1 |

**Figure S1.** Distribution of isolates by MIC differences between EUCAST and gradient concentration strips method (GCS) for itraconazole and posaconazole. ITZ: itraconazole; PCZ: posaconazole

***
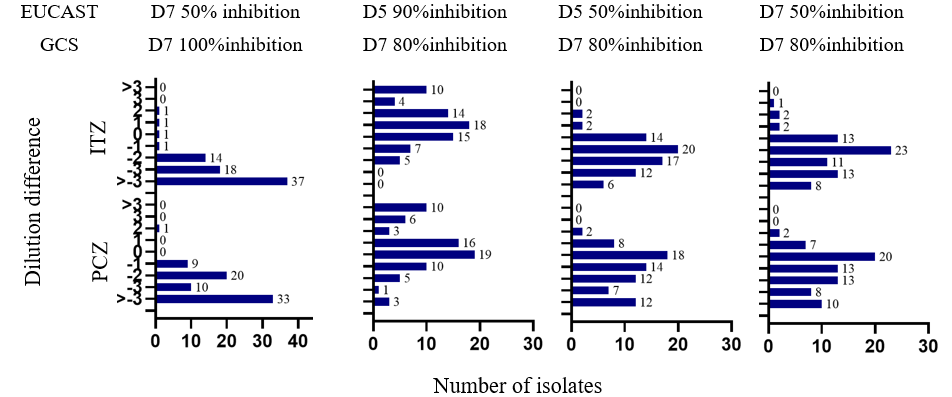
***
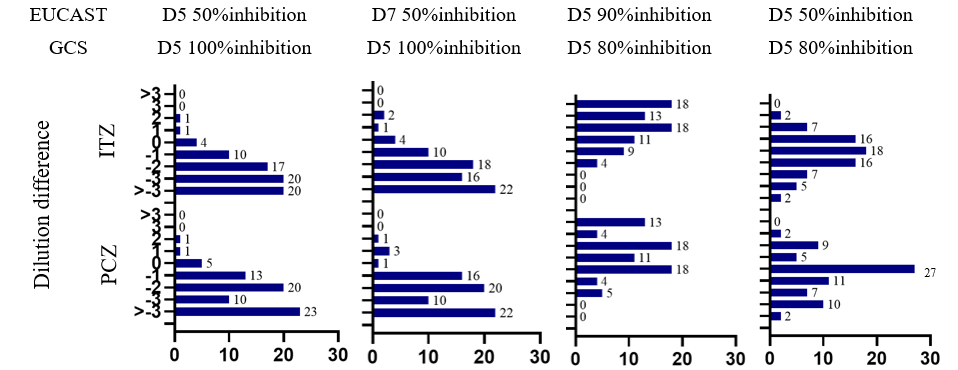


| ITZ | mean | -2.45 | -2.44 | 2.73 | -0.14 |
| --- | --- | --- | --- | --- | --- |
|  | median | -2.55(IQR : -3.57/-1.52) | -2.55 (IQR : -3.61/-1.52) | 2 (IQR : 1/3.39) | 0 (IQR : -1/1) |
| PCZ | mean | -2 | -2 | 2 | 0 |
|  | median | -2 (IQR : -3.55/-1) | -2 (IQR : -3.55/-1) | 1 (IQR : 0.41/2.41) | 0 (-1.52/0.41) |

| ITZ | mean | -3.42 | 1.52 | -1.35 | -1.34 |
| --- | --- | --- | --- | --- | --- |
|  | median | -3.55 (IQR : -4.57/-2.55) | 1 (IQR : 0.40/1.98) | -1.05 (IQR : -2.01/-0.52) | -1 (IQR : -2.55/-0.52) |
| PCZ | mean | -3 | 1 | -1 | -1 |
|  | median | -3 (-4.57/-2) | 0 (IQR : -0.58/1.95) | -1 (IQR : -2.55/0) | -1 (-2/0) |

***
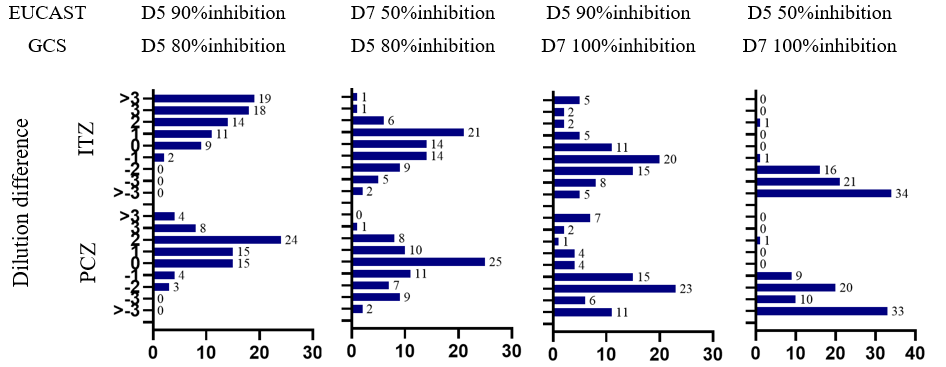
***

| ITZ | mean | 2.54 | -0.13 | -0.56 | -3.44 |
| --- | --- | --- | --- | --- | --- |
|  | median | 2.95 (IQR : 1.41/3.95) | 0 (IQR : -1/1) | -1 (IQR : -2 /-0.02) | -3 (IQR : -4.57/-2.55) |
| PCZ | mean | 1 | 0 | -1 | -3 |
|  | median | 1 (IQR : 0.41/2.37) | 0 (IQR : -1/0.96) | -2 (IQR :-2.01/-0.58) | -3 (-4.97/-2) |
